# The Roles of 2-Hydroxyglutarate

**DOI:** 10.3389/fcell.2021.651317

**Published:** 2021-03-26

**Authors:** Xin Du, Hai Hu

**Affiliations:** ^1^Guangdong Provincial Key Laboratory of Malignant Tumor Epigenetics and Gene Regulation, Sun Yat-sen Memorial Hospital, Sun Yat-sen University, Guangzhou, China; ^2^Department of Oncology, Sun Yat-sen Memorial Hospital, Sun Yat-sen University, Guangzhou, China

**Keywords:** 2-Hydroxyglutarate, epigenetics, metabolism, immunology, isocitrate dehydrogenase

## Abstract

2-Hydroxyglutarate (2-HG) is structurally similar to α-ketoglutarate (α-KG), which is an intermediate product of the tricarboxylic acid (TCA) cycle; it can be generated by reducing the ketone group of α-KG to a hydroxyl group. The significant role that 2-HG plays has been certified in the pathophysiology of 2-hydroxyglutaric aciduria (2HGA), tumors harboring mutant isocitrate dehydrogenase 1/2 (IDH1/2mt), and in clear cell renal cell carcinoma (ccRCC). It is taken as an oncometabolite, raising much attention on its oncogenic mechanism. In recent years, 2-HG has been verified to accumulate in the context of hypoxia or acidic pH, and there are also researches confirming the vital role that 2-HG plays in the fate decision of immune cells. Therefore, 2-HG not only participates in tumorigenesis. This text will also summarize 2-HG’s identities besides being an oncometabolite and will discuss their enlightenment for future research and clinical treatment.

## Introduction

**FIGURE 1:**
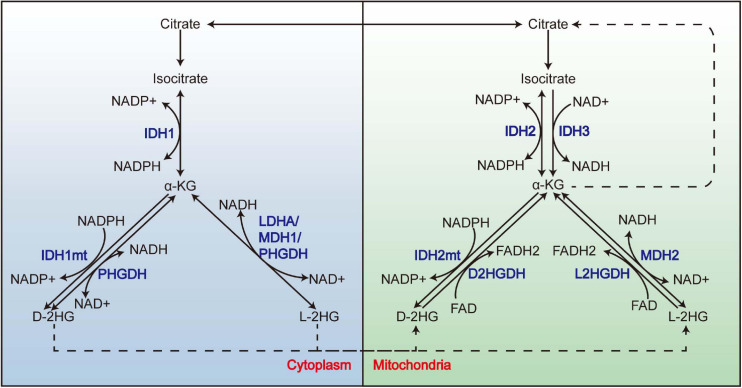
The promiscuity of LDHA, MDH, and PHGDH and the metabolism of 2-HG. 2-HG derives from the promiscuity of LDHA, MDH1, and PHGDH in the cytoplasm and MDH2 in the mitochondria. Mutations of IDH1 and IDH2 endow them the neomorphic enzyme activity to convert α-KG to D-2HG. L-2HG and D-2HG are removed by L2HGDH and D2HGDH from the mitochondria, respectively. LDHA, lactate dehydrogenase A; MDH, malate dehydrogenase; 2-HG, 2-hydroxyglutarate.

2-Hydroxyglutarate (2-HG) is structurally similar to α-ketoglutarate (α-KG), which is an intermediate of the tricarboxylic acid (TCA) cycle; it can be generated by reducing the ketone group of α-KG to a hydroxyl group. With its C-2 being a chiral carbon, it has two enantiomers: L-2HG and D-2HG. Besides the well-known IDH1/2mt, which acquires a neomorphic catalytic activity that reduces α-KG to the D enantiomer of 2-HG (Dang et al., 2010; Ward et al., 2010), 2-HG also derives from the “promiscuity” of some enzymes (Figure 1); it means that apart from their primary reaction, these enzymes can catalyze an “unwanted” reaction at a low rate. For example, besides the conversion between malate and oxaloacetate, malate dehydrogenase (MDH) also reduces α-KG to L-2HG, although the catalytic efficiency is 10^7^–10^8^-folds lower than its primary reaction (Rzem et al., 2007). Lactate dehydrogenase (LDH) A (LDHA) (Intlekofer et al., 2017) and SerA (Zhao and Winkler, 1996), the *Escherichia coli* homolog of PHGDH, was also reported to synthesize L-2HG. As for D-2HG, PHGDH also owns the property of producing D-2HG from α-KG (Fan et al., 2015). IDH2 catalyzes the reductive carboxylation of glutamine-derived α-KG, producing citrate and NADPH (Wise et al., 2011); this process is accompanied by an accumulation of D-2HG, although whether D-2HG derives from the catalysis of IDH2 directly remains uncharted. In respect of the clearance of 2-HG, the two enantiomers are removed by L2HGDH (Rzem et al., 2004) and D2HGDH (Achouri et al., 2004) in the mitochondria. Both enzymes utilize FAD as an electron acceptor to execute the oxidation of 2-HG, thereby generating α-KG and FADH2.

2-Hydroxyglutarate did not draw much attention after being identified; this continued until the discovery of 2-hydroxyglutaric aciduria (2-HGA) (Ye et al., 2018). 2-HGA is a rare disease characterized by an accumulation of 2-HG in body fluids; its clinical manifestations encompass a series of psychiatric and neurological symptoms such as epilepsy, and signs like hypotonia, macrocephaly, and extrapyramidal symptoms, along with neuroimaging changes (Kranendijk et al., 2012; Rodrigues et al., 2017). By which enantiomer accumulates in the body fluids, it can be classified into D-2HGA, L-2HGA, and “combined D,L-2-hydroxyglutaric aciduria” (D,L-2HGA) (Kranendijk et al., 2012). L-2HGA and the type I D-2HGA are derived from a mutation of L2HGDH and D2HGDH, respectively (Rzem et al., 2004; Struys et al., 2005); the etiology of the type II D-2HGA is the gain-of-function mutation of IDH2 (Kranendijk et al., 2010), and that of the D,L-2-HGA is the mutation of SLC25A1, which acts as a mitochondrial citrate carrier (Nota et al., 2013). The neurotoxic effect of 2-HG forms the pathophysiological basis of 2-HGA, since the accumulation of both enantiomers leads to impaired creatine kinase activity, induced oxidative stress, and a higher glutamate uptake into synaptosomes and synaptic vesicles (Junqueira et al., 2003, 2004; Kranendijk et al., 2012).

Although 2-HGA brought 2-HG to the attention of researchers, it was its identity as an “oncometabolite” made it famous. Two genomic sequencing studies published in 2008 and 2009 discovered the high mutation rate of IDH1 in glioblastoma and acute myeloid leukemia (AML), respectively (Parsons et al., 2008; Mardis et al., 2009). A series of researches after then demonstrated the existence of IDH1/2mt in 70–80% of grade II/III glioma and secondary glioblastoma, and also in about 20% of AML (Yan et al., 2009; Medeiros et al., 2017). Besides that, IDH1/2 mutation also consists in chondrosarcoma, osteosarcoma, cholangiocarcinoma, and prostate cancer (Gagné et al., 2017). These mutations mainly take place at the R132 of IDH1 and R172 of IDH2 (Ye et al., 2018). To date, D-2HG has been supposed to trigger oncogenesis by altering the methylation pattern of DNA and histone, expression and activity of DNA repair proteins, collagen maturation, and immune response (Gagné et al., 2017; Reiter-Brennan et al., 2018), although increasing attention is being paid to its antitumor effect (Fu et al., 2015; Su et al., 2018).

As for the other enantiomers, L-2HG, its accumulation has been reported in clear cell renal cell carcinoma (ccRCC), which resulted from the copy number loss of L2HGDH (Shelar et al., 2018). Increased L-2HG in ccRCC suppresses the methylcytosine dioxygenase (TET) and KDM6A, leading to hypermethylation of DNA and histone. On the other hand, overexpression of L2HGDH is issued in a lower L-2HG level and restored the altered epigenetic state, thus hindering the growth of ccRCC. Therefore, L-2HG is also regarded as an oncometabolite (Shim et al., 2014; Shelar et al., 2018).

2-Hydroxyglutarate is famous for its involvement in tumorigenesis; however, this just represents for its pathological effect. Then, does 2-HG function in other contexts? It is reported that L-2HG accumulates in hypoxia or acidic pH (Harris, 2015; Oldham et al., 2015; Nadtochiy et al., 2016); there are also researches confirming the significant role that 2-HG plays in the fate decision of immune cells (Tyrakis et al., 2016; Sun et al., 2020). It seems that 2-HG has physiological roles that extend beyond its being an oncometabolite; therefore, this text will also summarize 2-HG’s identities besides its being an oncometabolite and discuss their enlightenment for future research and clinical treatment.

## The Tumorigenic Effect of 2-Hydroxyglutarate

### The Oncometabolite 2-Hydroxyglutarate Inhibits α-Ketoglutarate-Dependent Dioxygenases

Transformation by IDHmt has been demonstrated in numerous researches. It was revealed that the mutation of IDH occurred earlier than the mutation of *TP53* or the loss of 1p/19q (Watanabe et al., 2009). It can also happen in the early stage of AML and be detected in a population of pre-leukemic stem cells in some patients. These facts suggest that IDH mutation represents an early event in gliomagenesis and leukemogenesis (Langemeijer et al., 2009; Corces-Zimmerman et al., 2014; Shlush et al., 2014). Introducing IDH1 R132H facilitated anchorage-independent growth of astrocytes lacking *CDKN2A*, *ATRX*, and *PTEN* (Modrek et al., 2017; Mukherjee et al., 2018; Philip et al., 2018), and the expression of IDH1/2 mutant impaired hepatocyte differentiation (Saha et al., 2014). Though it seems that IDHmt alone is not sufficient to induce tumorigenesis *in vivo* (Chaturvedi et al., 2013; Bardella et al., 2016; Philip et al., 2018), it cooperates with other oncogenes or deficiency of tumor suppressors to trigger tumorigenesis (Chaturvedi et al., 2013; Chen et al., 2013; Bardella et al., 2016; Philip et al., 2018). Discovering the neomorphic activity of IDHmt did shed light on the tumorigenic effects that IDHmt imposes. These mutations in the active site of IDH disturb the interaction between its binding pocket and the β-carboxyl of isocitrate (Dang et al., 2010; Ward et al., 2010). It was reported that IDH1 R132 substitution altered the spatial position of Y139, and then the hydroxyl moiety of Y139 occupied the space of isocitrate (Dang et al., 2010) and led to a closed conformation, thus favoring the binding of NADPH and the subsequent reduction of α-KG (Dang et al., 2010). IDH normally functions as a homodimer; the WT:MT heterodimer significantly decreases its catalytic ability compared with the WT:WT homodimer, because the two subunits alter their conformation in a co-operative way. The disability in interacting with isocitrate of the mutant subunit could impede the conformation change of the wild-type (WT) one (Zhao et al., 2009).

Elevation of L-2HG is observed in ccRCC; it takes glutamine as predominant carbon source and is generated by the promiscuous activity of MDH. L-2HG is removed by L2HGDH under physiological conditions, but a large portion of ccRCC (∼40%) undergo loss of heterozygosity (LOH) of 14q where *L2HGDH* locates in. What is more, proliferator-activated receptor gamma co-activator 1-alpha (PGC-1α), the transcription factor regulating L2HGDH expression, is usually downregulated in renal tumor cells (Brinkley et al., 2020). All of these results in L2HGDH downregulation and the subsequent accumulation of L-2HG. L2HGDH knockdown promoted malignant phenotypes, while its expression did the opposite; moreover, its under-expression is associated with poor prognosis (Shelar et al., 2018; Shenoy et al., 2019). Therefore, L2HGDH acts a tumor suppressor in ccRCC, and the accumulated L-2HG is thought to be an oncometabolite.

Both enantiomers of 2-HG were found to inhibit α-KG-dependent dioxygenases including TET2 and Jumonji-C (JmjC) histone demethylases by completely interacting with the α-KG-binding site (Chowdhury et al., 2011; Xu et al., 2011). These oxidases vary in their sensitivity to 2-HG; for example, the IC50 of histone demethylase JMJD2C to D-2HG is 79 ± 7 μM, while the value of hypoxia-inducible factor (HIF) is 1,500 ± 400 μM (Chowdhury et al., 2011). L-2HG is significantly more potent in inhibiting these enzymes than D-2HG (Chowdhury et al., 2011; Koivunen et al., 2012). For a clear inhibition on dioxygenases, a 100-fold molar excess of 2-HG over α-KG is required (Chowdhury et al., 2011); these facts suggest that D-2HG functions as a weak antagonist of α-KG. Under physiological conditions, the level of D-2HG is too low to significantly inhibit these enzymes. When IDH is mutant, α-KG is consumed to generate a concentration as high as 3–35 mM of D-2HG (Dang et al., 2010), which far exceeds the IC50 of these α-KG-dependent dioxygenases; this provides clues to detect the mechanism of the tumorigenic effect of D-2HG, and similarly of L-2HG.

### 2-Hydroxyglutarate and DNA Methylation

Profiling promoter methylation of the glioblastoma samples in The Cancer Genome Atlas (TCGA) identified a distinct subset with a hypermethylated phenotype, named glioma-CpG island methylator phenotype (G-CIMP). This phenotype exhibited a tight association with IDH1 mutation (Noushmehr et al., 2010). A similar phenomenon was also discovered in AML patients, and the IDH1/2 mutation exhibited an enrichment in patients with intermediate-risk cytogenetics (Figueroa et al., 2010). Subsequently, it was uncovered that the expression of IDH1mt could mirror the G-CIMP in low-grade glioma (Turcan et al., 2012), and IDH1/2mt was found to increase the global level of 5-mC (Figueroa et al., 2010). The mutation of IDH1 and IDH2 is mutually exclusive with each other (Figueroa et al., 2010), suggesting that they transform cells in a similar way; consistent with that, the methylation change is similar between IDH1mt and IDH2mt AML samples (Figueroa et al., 2010). These facts indicate that IDH mutation defines a specific epigenome and clinical features. According to this, the 2016 WHO brain tumor classification classified (anaplastic) astrocytoma based on the existence of IDH mutation, and the diagnosis of (anaplastic) oligodendroglioma requires coexistence of IDH mutation and 1p/19q co-deletion (Preusser and Marosi, 2017).

Now that IDH1/2 acquires a neomorphic enzyme activity of converting α-KG to 2-HG, D-2HG may play a vital role in the transformation by IDHmt. Indeed, D-2HG treatment recapitulated IDH1-induced differentiation block and cytokine-independence in hematopoietic cells (Losman et al., 2013). Similarly, D-2HG supplement promoted anchorage-independent growth in glioma cells (Philip et al., 2018). To investigate the tumorigenic mechanism of D-2HG and considering that D-2HG inhibits α-KG-dependent dioxygenases, a group transfected TF-1 human erythroleukemia cell line with a pool of shRNA targeting these enzymes. A relative enrichment of shRNAs targeting TET2 but not its paralog TET1 was detected after a period of culturing; TET2 knockdown recapitulated the transforming effect of IDH1mt and D-2HG in TF-1 cells, indicating that TET2 may mediate transformation induced by IDH1mt and D-2HG (Losman et al., 2013). Its activity converting 5mC to 5-hydroxymethylcytosine accounts for the association between IDHmt and a hypermethylated genome observed in AML and glioma samples. The co-expression of IDH1/2mt and the catalytic domain of TET1 or TET2 decrease 5-hmC to a nearly undetectable level, suggesting that IDHmt caused abnormal DNA methylation by inhibiting TETs (Xu et al., 2011). What is more, TET2 mutation is frequently observed in myeloid cancers, in about 24% of secondary AML and 22% of chronic myelomonocytic leukemia, while it is mutually exclusive with mutation in IDH (Delhommeau et al., 2009; Figueroa et al., 2010). In another study, hypermethylation of TET2 promoter was observed in a small fraction (16%) of low-grade diffuse gliomas without IDH mutation, but this phenomenon did not occur in gliomas with IDH mutation. The deficiency of TET2 induced a similar methylome with that IDHmt does in glioma (Figueroa et al., 2010). These facts all suggest that TET2 deficiency mediates the tumorigenic effects of mutant IDH and D-2HG. The abnormal *de novo* DNA methylation plays a vital role in tumorigenesis, repressing genes that are indispensable for differentiation (Klutstein et al., 2016). The infection of mutant IDH enriched 5-mC at the promoter of differentiation-associated genes and induced genes associated with stem cell (Figueroa et al., 2010; Turcan et al., 2012). Other modules can also be affected by the anomalous methylation; the hypermethylation of CTCF-binding sites comprises its binding with the insulator protein CTCF in glioma, permitting an enhancer to interact with *PDGFRA*, which is a glioma oncogene (Flavahan et al., 2016) and blocking differentiation by disassociating SOX2 from putative enhancers (Modrek et al., 2017). In conclusion, aberrant DNA methylation mediated the oncogenic effect of IDHmt and D-2HG.

L-2HG is a bona fide oncometabolite; its supplement promotes ccRCC cell line migration (Shelar et al., 2018). Both increased L-2HG and L2HGDH knockdown led to diminished 5-hmC level, which is strongly associated with tumor aggressiveness and an unfavorable prognosis, and restoring the activity of TET2 leads to inhibition of ccRCC growth both *in vitro* and *in vivo* (Shim et al., 2014; Shenoy et al., 2019). Therefore, the inhibition of L-2HG on TETs and the subsequent altered DNA methylation may mediate its oncogenic effect. It was reported that L-2HG upregulated PHLDB2 to promote vasculogenic mimicry (VM) in renal cancer cell lines in a way associated with DNA methylation; the DNA methyltransferase inhibitor counteracted with L-2HG in regulating PHLDB2 (Wang et al., 2020). Although its association with DNA methylation is well established, little is known about how L-2HG-induced DNA hypermethylation triggers tumorigenesis, so further investigation is required.

### 2-Hydroxyglutarate and Histone Methylation

The Jmjc histone demethylase is another member of the α-KG-dependent dioxygenase (Chowdhury et al., 2011), and it is sensitive to the perturbation of 2-HG. As reported, the mutant IDH2 or its product D-2HG compromised MyoD-driven differentiation in fibroblast cell line. Mechanistically, D-2HG induced H3K9me3 at those MyoD-binding loci, decreasing their accessibility to MyoD (Schvartzman et al., 2019). Likewise, IDH2mt expression or D-2HG treatment block adipocyte differentiation; this is correlated with an enrichment of the repressive mark H3K9me3 at some lineage-specific gene (Lu et al., 2012). Consistently, the expression of KDM4C, the demethylase for H3K9me3, is required during the differentiation of adipocyte. And it is interesting that observable changes in histone methylation preceded DNA methylation in the IDH1mt-expressing adipocyte. In another study, IDHmt inhibitor induced histone demethylation that predated DNA demethylation (Kernytsky et al., 2015). The above cases indicate that the methylation of histone is more sensitive to that of DNA in the face of D-2HG and acts as an independent factor in transformation. In line with this, inhibitors targeting IDH mutant induced differentiation by reversing the abnormal histone methylation at some genes, which are implicated in differentiation (Rohle et al., 2013; Kernytsky et al., 2015; Nakagawa et al., 2019).

As reported, L2HGDH overexpress decreased H3K27me3 and H3K9me3 in RCC cell line (Shim et al., 2014). Comparing the transcriptome of A498 RCC cell line overexpressing L2HGDH with the control group revealed that the upregulated genes showed an enrichment for the targets of polycomb repressor complex 2 (PRC2) and/or H3K27me3 target genes (Shelar et al., 2018). In accordance with that, the H3K27 histone demethylase KDM6A is frequently mutated in renal carcinoma (Dalgliesh et al., 2010), KDM6A knockdown recapitulates the malignant phenotypes that L-2HG induced, and PRC2 knockdown did the opposite. The above facts suggest that the L2HGDH/L2HG axis induces tumorigenesis through an aberrant histone methylation.

### 2-Hydroxyglutarate and Hypoxia-Inducible Factor Signaling

Hypoxia adaption is largely mediated by HIFs, which function as a heterodimer and consists of an α-subunit and a β-subunit. There are three families of HIF in human cells: HIF-1, HIF-2, and HIF-3. The α-subunit is oxygen-sensitive. In normoxia, it is hydroxylated by HIF prolyl hydroxylases (PHD/EGLN). Subsequently, the hydroxylated HIF-α is recognized by von Hippel–Lindau (VHL) E3 ligase for degradation through the proteasome pathway (Balamurugan, 2016). FIH can also hydroxylate HIF-α, resulting in its disability in interacting with transcription cofactors. In hypoxia, the activity of PHD and FIH is diminished. The stabilized HIF-α then translocated to nucleus and interact with the β-subunit, the HIF-1α/β heterodimer, and facilitates the transcription of its downstream targets (Balamurugan, 2016).

Although PHD, the enzyme hydroxylating HIF-1α, belongs to the α-KG-dependent dioxygenase family, the influence of 2-HG on HIF pathway seems debatable. In the research of Zhao et al. (2009) overexpression of IDH1mt or downregulation of the WT allele elevated the level of HIF-1α, while α-KG supplement diminished this effect; they inferred that IDH1 or its mutant allele regulated HIF-1α by controlling the level of α-KG. Although D-2HG was not discussed in this research, it can be concluded that expression of IDHmt upregulated HIF-1α. However, Koivunen et al. (2012) discovered that IDH1mt expression reduced HIF-1α level, because D-2HG acted as a co-substrate of EGLN, and this was enantiomer-specific. EGLN1 knockdown comprised the proliferation of IDH1mt astrocyte while downregulating HIF-1α did the opposite; these results suggest that IDHmt-derived D-2HG activates EGLN1, and subsequent downregulation of HIF-1α may lead to the tumorigenesis of glioma (Koivunen et al., 2012). Consistently, bioinformatic analysis of 288 low-grade diffuse or anaplastic gliomas samples from TCGA revealed that those with IDHmt displayed a significant inhibition on hypoxia-mediated signaling (Kickingereder et al., 2015). The discrimination between the two enantiomers in inhibiting EGLN1 was also discovered in hematopoietic cells. D-2HG rather than L-2HG could recapture the transforming effect of IDHmt expression, and this is related to its discrimination in inhibiting EGLN1 (Losman et al., 2013). It seems that, in astrocytes and hematopoietic cells, HIF may act as a tumor suppressor, and the mutation of IDH inhibits HIF signaling through D-2HG and promotes oncogenesis. The mechanism of D-2HG to promote EGLN also remains controversial. Contradicting the opinion that D-2HG acts as a co-substrate to activate EGLN1, another group demonstrated that both enantiomers could be oxidized to α-KG non-enzymatically through a Fenton-type chemistry mediated by iron and other physiologically relevant reducing agents such as L-ascorbate and GSH (Tarhonskaya et al., 2014); the α-KG derived from 2-HG oxidation is at levels to support catalysis by PHD. They attributed the result that PHD2 catalysis proceeded in the presence of D-2HG rather than L-2HG in the research of Koivunen P. to the more potent inhibition of L-2HG than D-2HG and/or the contamination of D-2HG by α-KG (Tarhonskaya et al., 2014). Further researches are required to explore the conditions dictating the influence of D-2HG on PHD and the mechanisms behind it.

### 2-Hydroxyglutarate and Other α-Ketoglutarate-Dependent Dioxygenases

The *Escherichia coli* protein AlkB was demonstrated to be an α-KG-dependent dioxygenase; it works as a DNA repair enzyme, oxidizing the alkyl groups in cytotoxic lesions 1-methyladenine (m1A) and 3-methylcytosine (m3C) induced by alkylation agents (Trewick et al., 2002; Chen et al., 2017). Its homolog in human was identified as the ALKBH family, including ALKBH1-8 and FTO. ALKBH2 and ALKBH3 play the major role in restoring the alkylated DNA (Chen et al., 2017). L-2HG and D-2HG are found to inhibit ALKBH2/3 in a pathophysiologically relevant level (Chen et al., 2017), restraining their activity in repairing m1A and m3C in ssDNA and dsDNA. DNA repair deficiency may help to accumulate mutations in the genome, thereby contributing to tumorigenesis; this is consistent with the fact IDH mutation is an early event in gliomagenesis and leukemogenesis (Langemeijer et al., 2009; Watanabe et al., 2009; Corces-Zimmerman et al., 2014; Shlush et al., 2014) as well as the higher mutation load in IDmt LGG compared with IDHwt LGG in TCGA database (Kohanbash et al., 2017).

By utilizing a brain-specific IDHmt knockin mouse model, Sasaki et al. (2012) discovered that mice expressing IDHmt exhibited a deficiency in collagen maturation. This phenomenon was related to the inhibition of D-2HG on prolyl-4-hydroxylases I, II, and III (C-P4H I–III), and procollagen-lysine 2-oxoglutarate 5-dioxygenases 1, 2, and 3 (Plod1–3), which mediated collagen hydroxylation. As hydroxylation is necessary for collagen maturation, basement membrane aberrations and hemorrhage were induced in mice with IDHmt tumor (Sasaki et al., 2012; Pellegatta et al., 2015). Mutation of genes encoding collagen is frequent in glioblastoma (GBM); what is more, IDHmt seldom coexists with basement membrane-related gene mutations (Sasaki et al., 2012). Therefore, collagen immaturation mediated by D-2HG may be involved in GBM tumorigenesis. As reported, an accumulation of immature procollagen-IV in endoplasmic reticulum (ER) led to ER stress and autophagy of ER, inhibiting the autophagic degradation of the ER-caused apoptosis (Viswanath et al., 2018b). Therefore, the attenuated collagen maturation and its downstream effect may be druggable in the context of IDH mutation.

### D-2-Hydroxyglutarate Modulates the Immune Microenvironment

**FIGURE 2:**
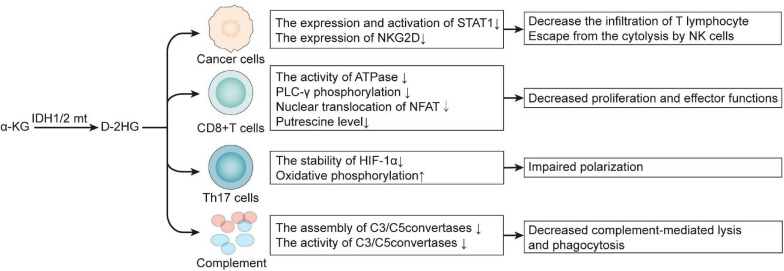
D-2HG derived from mutant IDH is an immunosuppressive metabolite. 2-HG, 2-hydroxyglutarate.

D-2HG is not only a cancer cell-autonomous oncometabolite, but it also contributes to an immunosuppressive milieu by exerting on immune cells both directly and indirectly (Figure 2). By inhibiting the expression and activation of STAT1, D-2HG attenuates chemokine CXCL10 secretion in glioma cell lines, thereby suppressing cytotoxic T lymphocyte infiltration in the tumor site (Kohanbash et al., 2017). Extracellular D-2HG is five-folds higher than the intracellular level; therefore, it can be inferred that tumor-infiltrating lymphocytes are faced with a high concentration of D-2HG (Böttcher et al., 2018; Bunse et al., 2018). Although D-2HG is poorly cell-penetrative, the transporter SLC13A3 can import D-2HG into T lymphocyte. An excess of D-2HG in T lymphocyte suppresses ATPase, subsequently reducing ATP and attenuating PLC-γ phosphorylation, which both resulted in decreased nuclear translocation of the nuclear factor of activated T cells (NFAT), which is of much concern in T lymphocyte cell activation. Moreover, D-2HG also diminished putrescine, which stimulated T lymphocyte cell proliferation (Bunse et al., 2018; Zhang et al., 2018). Apart from chemotaxis, activation, and proliferation, T lymphocyte differentiation is no exception. D-2HG impairs Th17 polarization by destabilizing HIF-1; this is enantiomer-specific since L-2HG did not exert such effect (Böttcher et al., 2018), although IDHmt glioblastoma expresses significantly lower PD-L1 compared with IDHwt (Wang et al., 2016), and there is another research revealing that D-2HG induces transient *PD-L1* promoter hypermethylation, thus lowering the expression of PD-L1 (Rover et al., 2018). On the whole, D-2HG suppresses antitumor T cell immunity. Inspired by this, targeting the mutant IDH may synergize with immunotherapy. Combining IDH1 inhibitor with anti-PD-1 improved prognosis in C57BL/6J mice bearing IDH1mt glioma (Bunse et al., 2018). The IDH inhibitor IDH-C35 significantly enhances CD8 + T cell infiltration and prolongs the survival of glioma-associated antigens vaccinated mice challenged with IDHmt glioma (Kohanbash et al., 2017). Tumor vaccine is another way of immunotherapy besides blocking the immune checkpoint, and the mutant IDH is quite a tumor-specific neoantigen. Vaccination of transgenic mice expressing human major histocompatibility complexes (MHCs) with a peptide in the IDH1mt induced a CD4 + T-dependent immune response suppresses the growth of the IDH1mt sarcoma in an MHC-humanized animal model (Schumacher et al., 2014). Consistent with this, another group immunized mice bearing intracranial glioma with peptides encompassing the IDH1 mutation site, which significantly prolonged the survival of mice bearing IDH1mt and even cured 25% of them (Pellegatta et al., 2015). Therefore, besides immune checkpoint inhibitors, IDHmt-derived peptide vaccination is also a prospective therapy, and their combination may find synergy with each other. IDH1 inhibitor amplified the efficiency of glioma-associated antigens vaccination, augmented the infiltration of CD8 + T cell and improved the survival of mice challenged with IDH1mt tumor (Kohanbash et al., 2017). And in the research of Bunse et al. (2018) adoptive transfer of T cells from IDH1R132H peptide-vaccinated C57BL/6J mice only works in the presence of IDH inhibitor.

Apart from adaptive immunity, D-2HG also limits the innate defense against tumor. Via D-2HG, IDHmt astrocyte inactivates complement, for it restrains the assembly of C5 convertase and inactivates assembled C3/C5 convertases in the classical pathway of complement activation; in the meantime, it inhibits the assembly of C3/C5 convertases in the alternative pathway (Zhang et al., 2018). By those means, the glioma cell ward off complement-mediated lysis and phagocytosis (Zhang et al., 2018). Natural killer (NK) cells are also executors in excluding tumor. The receptor NK group 2D (NKG2D) activates NK cells when ligating with NKG2D ligands (NKG2DLs) on the surface of targeted cells such as tumor cells (Zhang et al., 2016). IDHmt astrocyte cell lines and patient-derived glioma stem-like cells exhibit a lower expression of NKG2D than those with IDHwt, and this is associated with the hypermethylation of NKG2D promoters. Downregulating NKG2D helps tumor cells to escape cytolysis by NK cells, and the pretreatment of D-2HG confers IDHwt astrocytes resistance to that (Zhang et al., 2016), restoring NKG2DL expression by the DNMT inhibitor decitabine, which significantly prompted NK-induced cytotoxicity.

## 2-Hydroxyglutarate Production Confers Vulnerabilities

From the above, we can conclude that 2-HG triggers tumorigenesis through multiple mechanisms. However, its inhibition on α-KG-dependent dioxygenases and the altered epigenetic landscape also confers the tumor cells vulnerabilities (Table 1). For example, D-2HG sensitizes IDHmt malignancies to DNA damage. Chemotherapy combining procarbazine, CCNU/lomustine, and vincristine (PCV) attains a better prognosis in oligodendroglial patients harboring IDHmt compared with those with the WT allele (Cairncross et al., 2014). Mechanistically, among the three regimens in PCV therapy, CCNU and procarbazine are DNA alkylating agents, and the DNA repair enzymes ALKBH2/3 are inhibited by D-2HG, thereby sensitizing tumor cells to them (Wang et al., 2015). Also, the IDHmt expression gives rise to a “BRCAness” phenotype by impeding homologous recombination, rendering the tumor cells more susceptible to poly(adenosine 5′-diphosphate–ribose) polymerase (PARP) inhibitors (Sulkowski et al., 2017). ATM, encoded by the ataxia telangiectasia mutated gene (*ATM*), is also a DNA repair enzyme involved in sensing the double-strand break (DSB) and the mobilization of downstream repair machinery (Inoue et al., 2016). D-2HG attenuates the transcription of *ATM* by increasing the repressive mark H3K9me3 at its promoter, making for a susceptibility to DNA damage (Inoue et al., 2016; Molenaar et al., 2018). In addition, the inhibition of D-2HG on histone demethylase KDM4B brings about a hypermethylated state around DNA break loci; this masks the H3K9me3 signals required for homology-dependent repair (HDR) factor recruitment. KDM4B overexpression rescues the DNA repair deficiency in IDH mutant cell but not in IDH WT cells (Sulkowski et al., 2017; Sulkowski et al., 2020). In general, D-2HG suppresses DNA repair; as a consequence, synthetic lethal interaction can be established between IDHmt and DNA damage agents.

**TABLE 1:** The production of D-2HG confers vulnerability to IDHmt tumor.

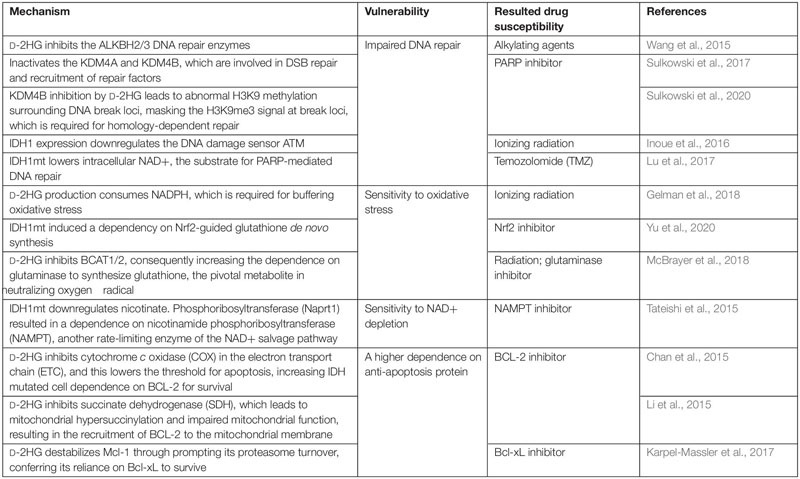

The process of 2-HG generation largely reprograms the metabolic landscape. To sum up, 2-HG synthesis consumes the reducing equivalent NADPH and NADH (Gilbert et al., 2014; Gelman et al., 2018; Hollinshead et al., 2018; Philip et al., 2018); downregulates lipid synthesis (Gelman et al., 2018; Viswanath et al., 2018a,b); reduces glycolysis (Chesnelong et al., 2014); stimulates glutamine metabolism (Reitman et al., 2014; Hollinshead et al., 2018; McBrayer et al., 2018); depletes the TCA flux; and impairs mitochondrial respiration (Reitman et al., 2011; Izquierdo-Garcia et al., 2015; Li et al., 2015; McBrayer et al., 2018). Some metabolic phenotypes among these confer IDHmt tumor vulnerabilities that can be exploited. 2-HG synthesis imposed an NADPH deficit, attenuating the ability to buffer oxidative stress. Both colorectal carcinoma and astrocyte cell lines expressing IDH1/2mt exhibited a hypersensitivity to ionizing radiation or H_2_O_2_ compared with those with IDH1/2wt (Gelman et al., 2018). Moreover, IDH1 glioma relies on nuclear factor (erythroid-derived 2)-like 2 (Nrf2)-governed glutathione synthesis pathway to maintain the intracellular redox homeostasis. Inhibiting Nrf2 by triptolide, a diterpenoid epoxide from *Tripterygium wilfordii*, compromised glutathione synthesis and caused synthetic lethality in IDHmt glioma by inducing oxidative damage (Yu et al., 2020). The vulnerability to oxidative stress also derives from the competitive inhibition of D-2HG on BCAT1/2, which lowers glutamate and prompts a dependence on the glutaminase to generate glutamate and downstream glutathione, which functions in neutralize intracellular reactive oxygen species (ROS). Hence, ablating glutaminase in IDHmt glioma extensively sensitizes cells to oxidative damage and radiation (McBrayer et al., 2018). In conclusion, the production of D-2HG is accompanied by an accessorial sensitivity to oxidative stress, and multiple layers of its antioxidant system can become druggable.

The expression of IDHmt reduces intracellular NAD+ by downregulating nicotinate phosphoribosyltransferase (Naprt1) in the NAD+ salvage pathway; a further depletion of NAD+ by targeting nicotinamide phosphoribosyltransferase (NAMPT), another enzyme in the metabolic pathway, induced a synthetic lethality with IDH mutation (Tateishi et al., 2015). Since NAD+ is a substrate for base excision repair (BER) mediated by PARP DNA repair machinery, there is a hardship for the IDHmt glioma to sustain the genomic integrity; this explains for the hypersensitivity of glioma with IDHmt to PARP inhibitors, which could synergize with a DNA alkylating agent such as temozolomide (TMZ) (Lu et al., 2017; Philip et al., 2018). D-2HG also inhibits the mitochondrial electron transport chain (ETC) by compromising the activity of cytochrome *c* oxidase (COX), resulting in a lower mitochondrial threshold to induce apoptosis, which sensitized those AML cells with IDH1mt to the inhibitor of the anti-apoptotic protein BCL-2 (Chan et al., 2015). Similarly, in another study, IDHmt-derived D-2HG inhibits succinate dehydrogenase (SDH), the accumulated succinate hypersuccinylate mitochondria, and finally induces the mitochondrial membrane localization of BCL-2 (Li et al., 2015). Another member of the BCL-2 family, Mcl-1, is downregulated in IDH1mt glioma cells, inducing a dependence on the anti-apoptosis protein Bcl-xL and thus the synthetic lethality of IDH1mt with Bcl-xL inhibitor (Karpel-Massler et al., 2017). These researches indicate that IDHmt has a specific role in dictating cell death program, inducing a dependence on certain anti-apoptotic protein, which is a potential target.

## D-2-Hydroxyglutarate Exerts Toxicity on Cancer Cell

Although 2-HG is identified as an oncometabolite, which facilitates tumor initiation, there is a paradox that IDHmt foreshadows a benign prognosis in glioma, and a similar trend also exists in AML (Parsons et al., 2008; Yan et al., 2009; Marcucci et al., 2010; Chou et al., 2011). Then, does 2-HG also exert toxicity on cancer cells? In the research of Fu et al. (2015) both enantiomers of 2-HG and the expression of IDHmt inhibited ATP synthase, thereby inactivating mTOR and cell growth. This uncovered the growth-suppressive effect of 2-HG on IDHmt glioma and suggested that there likely exist other mechanisms by which 2-HG exerts its potential toxicity on cancer cell. Rui Su et al., reported that exogenous D-2HG inhibited FTO, a RNA demethylase, which caused a decrease in *MYC* and *CEBPA* transcripts, suppressing tumor growth through the FTO/m6A/MYC/CEBPA axis. Exogenous L-2HG and overexpressed IDH1mt both recapitulated these effects (Fu et al., 2015). Consistent with this, in a subsequent study of the same group, targeting FTO induced a similar effect on FTO/m6A/MYC/CEBPA axis signaling through the same mechanism, leading to cell-cycle arrest in cancer stem cells (Su et al., 2020). Although D-2HG blunts immunity as a whole, it was reported that FTO inhibition decreased the expression of immune checkpoints like PD-L1, PD-L2, and LILRB, thereby counteracting the immune evasion of leukemia cells. Besides, D-2HG hinders neutrophil chemotaxis, which helps to establish an immunosuppressive ecology in the tumor microenvironment (TME) (Amankulor et al., 2017). Finally, IDHmt may induce susceptibility to some treatments. The above factors may be a proper explanation for the benign prognosis in glioma and AML with IDHmt; however, the inherent difference in genotypic milieu should not be neglected.

## The Roles of 2-Hydroxyglutarate Beyond Oncometabolite

### L-2-Hydroxyglutarate Takes Part in the Adaption to Hypoxia

It was revealed that multiple cell lines displayed an increase of 2-HG under hypoxia (Intlekofer et al., 2015; Oldham et al., 2015). L2HGDH but not D2HGDH knockdown decreased its concentration, indicating that L-2HG builds the majority of 2-HG pool; the result of gas chromatography–mass spectroscopy (GC-MS) also confirmed so. The sensitivity of L-2HG concentration to hypoxia varies among cell lines; L-2HG in C2C12 cells was only induced twofold (about 3 μM) by 2% oxygen (Chakraborty et al., 2019), which was a drop in the bucket compared with 304 ± 81 μM in SF188 cells by 0.5% oxygen (Intlekofer et al., 2015). Besides, not all α-KG-dependent dioxygenases are susceptible to the perturbation of L-2HG in hypoxia; the research of Chakraborty et al. (2019) revealed that the inhibition of KDM6A in C2C12 cells under hypoxia was directly resulted from change in oxygen concentration rather than the effect of HIF or L-2HG. The glutamine-derived α-KG builds the primary source of L-2HG, and the enzymes manufacturing L-2HG in hypoxia are LDHA and MDH; they consume α-KG and NADH to produce L-2HG and NAD+ (Intlekofer et al., 2017). Some studies reported that LDHA had a greater contribution in generating L-2HG than MDH (Intlekofer et al., 2015), while there are also others that demonstrated the opposite (Oldham et al., 2015); maybe it depends on cell types. When it comes to the mechanism of L-2HG’s accumulation, when there is a paucity of oxygen, some researchers think that the increased α-KG level and NADH/NAD+ ratio skew the reaction LDHA and MDH catalyzes toward the direction of L-2HG synthesis (Oldham et al., 2015), and downregulation of L2HGDH in hypoxia contributed to that too. An acidic intracellular environment resulted from oxygen limitation, which also favors the “promiscuous” reaction catalyzed by LDH and MDH, because an acidic pH sustains a protonated form of α-KG that binds to the substrate-binding pocket of LDHA more tightly via forming a hydrogen bond (Nadtochiy et al., 2016; Intlekofer et al., 2017). An analogous mechanism may be involved in the interaction between α-KG and MDH under acidic pH.

As described above, intracellular L-2HG elevates in the face of low-oxygen condition; it helps in alleviating hypoxia-induced reductive stress. Reductive stress is defined as an excess accumulation of reducing equivalents (specifically NADH, NADPH, and GSH) that exceeds the capacity of endogenous oxidoreductases (Xiao and Loscalzo, 2020). The oxidation of NADH by complex I is suppressed under hypoxia; this results in its accumulation, offering more electrons for one-electron reduction of oxygen that generates O_2_•- (Kussmaul and Hirst, 2006; Yang and Kahn, 2006; Clanton, 2007). Mechanisms by which reductive stress damages cells include disturbing signal transduction by ROS, inducing ER stress, and so on (Xiao and Loscalzo, 2020). In this process of reducing α-KG by NADH, L-2HG acts as a reservoir of excess reducing equivalents. L2HGDH knockdown not only mitigated mitochondrial membrane hyperpolarization but also impeded the generation of mitoSOX (Oldham et al., 2015), indicating that L-2HG metabolism was implicated in maintaining redox balance. It is explicit that being an α-KG-dependent dioxygenase, PHD, the key enzyme in regulating the stability of HIF-1α, can be suppressed by L-2HG. The IC50 value for the L form of 2-HG to PHD is 419 ± 150 μM (Chowdhury et al., 2011), which is comparable with the concentration of L-2HG quantitated in SF188 cells (304 ± 81 μM) under hypoxia. In accordance with that, downregulating L2HGDH in aerobic condition stabilized HIF-1α (Oldham et al., 2015). Therefore, L-2HG may assist in cell’s adaption to hypoxia via sustaining the activity of HIF pathway.

In summary, L-2HG partakes in acclimatizing hypoxia by regulating redox homeostasis and the activity of HIF pathway. Now that PHD can be inhibited by L-2HG, it is probable that KDM and TET are also sensitive to the perturbation of L-2HG, owing to their lower IC50. A study reported that hypoxia-induced L-2HG could inhibit KDM4C, thereby promoting the methylation of H3K9 (Intlekofer et al., 2015). Although its downstream effect remains obscure, it does suggest that besides sensing oxygen directly, TET and KDM4C can sense changes in oxygen concentration indirectly through L-2HG. In terms of D-2HG, although a study revealed a concomitant increased synthesis of D-2HG associated with IDH2-dependent carboxylation of α-KG (Wise et al., 2011), there is no definitive evidence demonstrating D-2HG’s contribution in the adaption to hypoxia. A conceivable explanation is that D-2HG elevates marginally relative to L-2HG; more importantly, D-2HG functions as a weak antagonist of α-KG-dependent dioxygenase, which is far less efficient than L-2HG (Xu et al., 2011).

### 2-Hydroxyglutarate Is an “Immunometabolite”

Accumulated studies suggest that there is a close relationship between metabolism and the activation, differentiation, and exhaustion of immune cells (Zhang and Romero, 2018; Yerinde et al., 2019). Some subsets of T cell exhibit a distinct metabolic feature. For example, naïve CD8 + T cells metabolically rely on oxidative phosphorylation, and their activation leads to a metabolic switch to glycolysis, while memory T cells heavily depend on fatty acid oxidation (Yerinde et al., 2019). Multiple processes of CD8 + T cells such as clonal expansion and memory formation are sustained by a distinct metabolic state (Yerinde et al., 2019). Such association between metabolism and function also exists in other subsets of immune cells. What is more, the function states of immune cells are also shaped by epigenetics (Gray et al., 2017). Now that metabolism and epigenetics have such a huge impact on immune cells, the metabolites linking metabolism and epigenetics such as 2-HG, fumarate, and acetyl-CoA are predicted to be vital in immune cells. Next, we are going to discuss the role of 2-HG in immune cells.

As mentioned in the research of Tyrakis et al. (2016) CD8 + T cell produced millimolar L-2HG in response to TCR triggering, resulting in an enrichment of active H3K4me3 mark and RNA pol II at *CD62L*, polarizing it to memory subsets. Moreover, accumulating L-2HG prompted a global increase of H3K27me3 and 5mC and knockdown of H3K27me2/3 demethylase Utx; and TET2 also motivated the expression of CD62L, indicating that epigenetic inhibition somewhere else in the genome triggers the transcription of *CD62L*. Although it was a little regrettable that the study did not identify these genes, it unveiled L-2HG’s identity of “immunometabolite” unprecedentedly, which mattered a lot in the fate decision of CD8 + T lymphocytes through a metabolic–epigenetic axis. A recent study further revealed that with Utx bound directly to *Prdm1*, through reduced demethylation of histone H3K27me3, its deficiency hampers the transcription of *Prdm1*-encoding Blimp1 that is an effector-associated transcription factor, triggering a memory-like phenotype (Yamada et al., 2019). Therefore, L-2HG dictates CD8 + T lymphocyte fate through the metabolism–epigenetics axis. This also uncovered the suppression on T lymphocyte in the TME from another aspect, since the carbon source of L-2HG, glutamine, is also taken as the primary nutritional source by multiple kinds of tumor.

Similar to L-2HG, D-2HG has also been demonstrated to be a mediator in T lymphocyte differentiation. In Tao Xu’s study, differentiating Th17 cells sustains a ∼5–10-fold higher concentration of D-2HG than did iTreg cells, inducing a hypermethylated state of *Foxp3* loci, which is a master transcription factor in the charge of Treg differentiation. ^15^N-labeling analysis demonstrated that D-2HG was mainly derived from glutamine, and glutamate oxaloacetate transaminase 1 (GOT1) inhibition reprogrammed Th17 differentiation toward iTreg cells (Xu et al., 2017). Similar with this, in the study of Licheng Sun, PM2.5 and indeno[1,2,3-*cd*]pyrene (IP) activated the expression of GOT1 in an AhR-dependent manner, thereby enhancing the metabolic flux from glutamine to D-2HG, bringing about a hypermethylated state of *Foxp3* loci and finally disrupted Th17/Treg ratio (Sun et al., 2020). This AhR-GOT1-D-2HG axis functions in the pathogenesis of asthma. By adopting a GOT1 inhibitor, they alleviated pulmonary inflammation in a mouse asthma model successfully.

We can conclude from the aforementioned studies that 2-HG interferes with T cell differentiation through epigenetics; they also inspire us the feasibility of regulating immune functions through the metabolism–epigenetics axis in T cells. Besides asthma, the imbalance of Th17/Treg ratio happens in a set of autoimmune diseases including psoriasis, inflammatory bowel disease, and multiple sclerosis (Lee, 2018); a similar role of D-2HG in their pathophysiology may exist. Other than modulating immune homeostasis in these diseases through drug intervention, adoptive immunotherapy is another viable choice. For instance, compared with injecting Th17 cells with control shRNA, adoptive transfer using Th17 cells infected with virus containing shGot1 significantly tempered disease severity in a mouse experimental autoimmune encephalomyelitis model (Xu et al., 2017). As regards oncotherapy, a pretreatment of L-2HG significantly exaggerated the persistence and antitumor capacity of adoptively transferred CD8 + T lymphocytes (Tyrakis et al., 2016). Therefore, benefiting from the plasticity of T lymphocyte differentiation and its reliance on epigenetics and metabolism (Kleinewietfeld and Hafler, 2013; Lee, 2018), we can take 2-HG as an entry point in maintaining the balance of immunity.

## Conclusion

During the recent 10 years, much efforts have been paid in exploring the pathophysiology of IDHmt, leading to remarkable achievements. Due to their excellent performance in clinical trial, the IDH inhibitors ivosidenib and enasidenib are approved by Food and Drug Administration (FDA) to treat adults with relapsed and refractory AML with IDH1/2 mutation. However, the application of IDHmt inhibitors in solid tumor seems not that effective; to crack this hard nut, a deeper insight on the relationship between IDHmt and the mutational background as well as TME is required. Besides targeting IDH directly, an alternative way is to exploit the vulnerabilities it confers, and this may be a more prospective strategy in the condition that IDHmt gradually becomes a passenger mutation from driver mutation (Waitkus et al., 2018). It is noteworthy that this strategy is incompatible with IDH inhibitor, as it depends on the activity of IDHmt. Until now, little is known with regard to the detailed tumorigenic mechanism of L-2HG, and this requires further investigation. As L-2HG is structurally analogous to D-2HG, they may share some common mechanisms such as suppressing α-KG-dependent dioxygenases. L-2HG generates in acidic environment and participates in the adaption to hypoxia. Does this also function in solid tumor, thus representing for the tumorigenic property of L-2HG from another perspective? Because the TME is quite a hypoxia and acidic milieu, L-2HG may help these areas establish an adaption to limited oxygen supply and, at the same time, generate phenotype heterogeneity, which contributes to variant therapeutic responses in the intratumoral religion through epigenetics and other mechanisms. It is surprising that in addition to oncometabolite, 2-HG also serves as immunometabolite. How the 2-HG metabolism in T lymphocyte will alter in the TME and what consequences this will bring about are worthy of exploration. What is more, does endogenous 2-HG regulate the functions of other immune cell subsets? Recently, L-2HG is demonstrated to promote axon regeneration in the central nervous system (Li et al., 2020). Therefore, 2-HG may also function in other physiological processes.

In conclusion, besides triggering tumorigenesis, 2-HG plays a significant role in adaption to hypoxia and acts as an “immunometabolite.” 2-HG is just one of the numerous metabolites; its functions reflect a tight association between metabolism and epigenetics, signaling transduction and other cellular processes, and these are all the themes of present researches on metabolism.

## Author Contributions

XD wrote the manuscript and made the figures. HH provided direction to the review. Both authors contributed to the article and approved the submitted version.

## Conflict of Interest

The authors declare that the research was conducted in the absence of any commercial or financial relationships that could be construed as a potential conflict of interest.
